# Individual identification of bony fishes using unique body markings: Implications and applications

**DOI:** 10.1111/jfb.70180

**Published:** 2025-08-20

**Authors:** Katie Dunkley, Samuel R. Matchette, Cheuk Yu Hau, Christian Drerup, Roxanne B. Holmes, Yvonne Sadovy de Mitcheson, James E. Herbert‐Read

**Affiliations:** ^1^ Christ's College University of Cambridge Cambridge UK; ^2^ Department of Zoology University of Cambridge Cambridge UK; ^3^ Department of Biology University of Oxford Oxford UK; ^4^ Swire Institute of Marine Science, School of Biological Sciences The University of Hong Kong Hong Kong SAR China; ^5^ Department of Biosciences Durham University Durham UK; ^6^ IUCN Groupers & Wrasses Specialist Group

**Keywords:** behaviour, conservation, ecology, individual recognition, management, photo‐identification

## Abstract

The natural variation in animal body markings, such as spots, stripes and blotches, offers a powerful tool for researchers, conservationists, citizen scientists and resource managers to distinguish specific individuals within species. By building libraries of photo‐identified individuals, we can track and differentiate individuals over time and space, thereby non‐invasively and often cost‐effectively studying species' biology and behaviour. This, in turn, enhances our understanding of species' ecological roles, and informs and supports effective conservation strategies. Although photo‐identification has been successfully applied to various aquatic species, including cetaceans, sharks and rays, it remains surprisingly underutilised for bony fishes. Indeed, despite their striking diversity of colours and patterns, relatively few studies have used natural markings to non‐invasively investigate bony fish biology and conservation. In this review, we highlight the potential of photo‐identification as a valuable research and management tool for these fishes in both field and laboratory environments. We outline the scientific, practical and ethical benefits of this approach, illustrating how individual identification can advance our understanding of fish biology and support their conservation efforts. We also discuss previous applications of photo‐identification in bony fishes, examine barriers to its broader adoption and address common misconceptions that may limit its use. We propose strategies to overcome these challenges driven by advancements in camera technology and artificial intelligence, and discuss scenarios where photo‐identification may prove particularly effective. Through this review, we therefore aim to encourage the broader use of natural body markings as a non‐invasive method in bony fish research, management and conservation.

## 1 INTRODUCTION

Tracking individuals across time and space holds significant implications for understanding species behaviour, life history, population dynamics, habitat use and the ecological roles they play within their environments (Costa‐Pereira et al., 2022; Kays et al., 2015; Stillman et al., 2015). Traditional tagging methods, like identification or electronic tags, have been used for decades to follow individuals throughout their lives and environments to collect individual‐level data. However, these methods are often invasive, posing risks to the health and survival of tagged individuals (Jepsen et al., 2015; Silvy et al., 2012; Soulsbury et al., 2020). Fortunately, many animals display a wide array of natural body markings, such as spots, blotches or stripe patterns (Miyazawa, 2020; Protas & Patel, 2008), which vary among individuals. These unique markings offer a non‐invasive alternative for distinguishing and tracking individuals over time and across different contexts.

**FIGURE 1:**
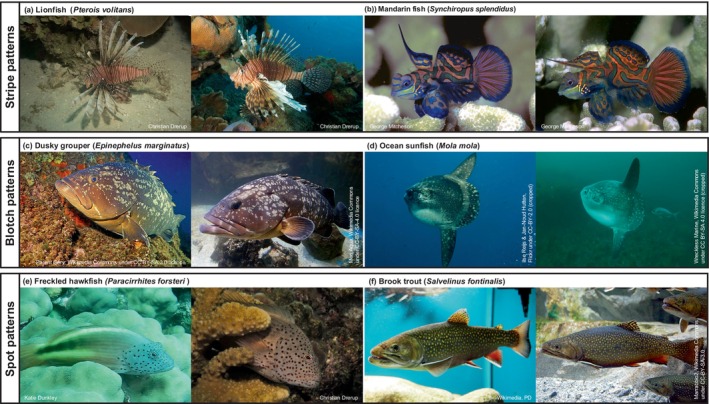
Example images of bony fishes that can be individually discriminated based on their body markings. Examples link to published species in Table 1, and for some species (e.g., mandarin fish), a mixture of markings (e.g., stripes with spots) can be used for identification (Sadovy de Mitcheson et al., 2022).

Photographs and videos have significantly enhanced researchers' ability to utilise natural markings for identifying and tracking individual animals. As a result, photo‐identification is becoming an increasingly important tool for tracking and studying a wide range of taxa, from large vertebrates (e.g., mammals: Barlow et al., 2011; Hiby et al., 2009; Urian et al., 2015, elasmobranchs: Arzoumanian et al., 2005; Marshall et al., 2011) to smaller vertebrates (e.g., reptiles: Rocha et al., 2013, amphibians: Gould et al., 2021; Schulte et al., 2024, bony fishes: Table 1; Figure 1). This method has even been applied to small invertebrates like sea stars (*Pisaster giganteus*, Chim & Tan, 2012) and beetles (*Rosalia alpina*, Caci et al., 2013). As a result, photo‐identification has expanded our understanding of species' ecology, evolution and behaviour (Armstrong et al., 2019; Holmberg et al., 2009; Sannolo et al., 2016; Urian et al., 2015), and has even contributed to their management, handling and protection (e.g., Hau & Sadovy de Mitcheson, 2019; Hiby et al., 2009; Zion, 2012).

**TABLE 1:** Non‐exhaustive list of previous studies and their research themes that have used unique body markings for individual identification of bony fishes.

Recognition feature	Species	References
Proof of concept: is photo‐identification a reliable technique for distinguishing individuals?		
Stripes	Cobitid fish (*Lefua echigonia*)	Akada et al., 2005
	Striped sorubim (*Pseudoplatystoma magdaleniatum*)	Lozano et al., 2017
	Lionfish (Figure 1a) (*Pterois volitans*)	Chaves et al., 2016
	Sumatra barb (*Puntigrus tetrazona*)	Bekkozhayeva et al., 2021
	Rabbitfish (*Siganus javus*)	Perrig & Goh, 2008
Blotches	Dusky grouper (Figure 1c) (*Epinephelus marginatus*)	Lelong, 1999
	Moray eel (*Gymnothorax moringa*)	Sebe, 2021
	Giant sunfish (*Mola alexandrini*)	Nyegaard et al., 2023
	Ocean sunfish (Figure 1d) (*Mola mola*)	Kushimoto et al., 2022
	Worm pipefish (*Nerophis lumbriciformis*)	Monteiro et al., 2014
	Giant sea bass (*Stereolepis gigas*)	Love et al., 2018
Spots	Patagonian catfish (*Hatcheria macraei*)	Barriga et al., 2015
	Cutthroat trout (*Oncorhynchus clarkii lewisi*)	Giford & Mayhood, 2014
	Chinook salmon (*Oncorhynchus tshawytscha*)	Merz et al., 2012
	Freckled hawkfish (Figure 1e) (*Paracirrhites forsteri*)	McInnes et al., 2020
	Atlantic salmon (*Salmo salar*)	Cisar et al., 2021; Stien et al., 2017
	Rabbitfish (*Siganus guttatus*)	Perrig & Goh, 2008
Other – bony plate	Loricariid (*Rineloricaria aequalicuspis*)	Dala‐Corte et al., 2016
Other – head shape	Seahorses (*Hippocampus guttulatus*, *Hippocampus hippocampus*)	Correia et al., 2021
Other – scales, lateral‐line shape	Common carp (*Cyprinus carpio*)	Bekkozhayeva & Cisar, 2022; Huntingford et al., 2013
	European seabass (Figure 2c) (*Dicentrarchus labrax*)	Bekkozhayeva & Cisar, 2022
Other – scars	Goliath grouper (*Epinephelus itajara*)	Giglio et al., 2014
Population dynamics: mark recapture		
Blotches	Pike (*Esox lucius*)	Kristensen et al., 2020
Spots	Seahorse (*H. guttulatus*)	Correia et al., 2014
	Weedy sea dragon (*Phyllopteryx taeniolatus*)	Martin‐Smith, 2011
	Brook trout (Figure 1f) (*Salvelinus fontinalis*)	Haxton, 2021
Space use: residency, movement and home‐range patterns		
Stripes	Broad‐barred goby (*Gobiodon histrio*)	Wall & Herler, 2009
Blotches	Dusky grouper (*Epinephelus marginatus*)	Desiderà et al., 2021
	Stoplight parrotfish (*Sparisoma viride*)	van Rooij et al., 1996
Behavioural interactions (e.g., territoriality, spawning)		
Stripes & spots	Mandarin fish (Figure 1b) (*Synchiropus splendidus*)	Sadovy de Mitcheson et al., 2022
	Tompot blenny (Figure 2b) (*Parablennius gattorugine*)	Naylor et al., 2023; Naylor & Jacoby, 2016
Blotches	Stoplight parrotfish (*S. viride*)	van Rooij et al., 1996
Conservation: improving enforcement of trade restrictions for threatened/CITES‐listed species		
Stripes	Napoleon wrasse (Figure 2a) (*Cheilinus undulatus*)	Hau & Sadovy de Mitcheson, 2019
Indirect evidence of individual‐level distinguishable markings^a^		
Stripes	Ambon damselfish (Figure 2c) (*Pomacentrus amboinensis*)	Siebeck et al., 2010
	Crown butterflyfish (*Chaetodon paucifasciatus*)	Levy et al., 2014
	African cichlid (*Neolamprologus pulcher*)	Kohda et al., 2015; Saeki et al., 2018
	Discus fish (*Symphysopdon aequifasciatus*)	Satoh et al., 2016
Blotches	Guppy (*Poecilia reticulata*)	Sogawa et al., 2023

^a^ Studies have identified individual differences in body patterning and shown individuals can discriminate one individual from another based on these visual markings (e.g., in the context of mate choice, dear neighbour, cooperative breeding).

Despite its broad potential, the use of photo‐identification has been somewhat biased towards certain taxa and remains underutilised in cases where it could be valuable. In the aquatic environment, for example, photo‐identification of scars, nicks, notches and colour patterns on individual cetaceans was used in the 1950s to provide new insights into dolphin habitat use, movements and life‐history characteristics (Caldwell, 1955; Urian et al., 2015). Photo‐identification then expanded to elasmobranchs in the 1990s (Marshall & Pierce, 2012). In 2012, Marshall and Pierce published an extensive review detailing the methods and promise of photo‐identification as a tool for studying sharks and rays. With over 150 citations to date (SCOPUS), this review has facilitated further photo‐identification studies for a number of elasmobranch species (e.g., great hammerhead shark, *Sphyrna mokarran*; Guttridge et al., 2017, bluespotted ray, *Neotrygon orientalis*; Sherman et al., 2018 and spotted wobbegong sharks, *Orectolobus maculatus*; Lee et al., 2014). Although Marshall and Pierce focused on elasmobranchs, the assumptions and potential of photo‐identification they highlight are readily transferable to other clades, such as the diversity of body markings and colourations observed on bony fishes (Miyazawa, 2020; Salis et al., 2019). However, despite the wide variation in markings across individuals in the bony fishes (Box 1), and the fact that their species diversity rivals that of all other vertebrates combined (Amundsen, 2003), the adoption of photo‐identification for these animals has been somewhat limited (Table 1). In fact, although published in the *Journal of Fish Biology*, Marshall and Pierce's review has been cited in studies applying photo‐identification to bony fishes only 10 times (10/151 citations as of July 2025, via SCOPUS) compared to 13 citations for terrestrial species, such as amphibians and insects (13/151), and 92 citations relating to elasmobranchs (92/151). With 35,700 fishes currently listed on FishBase (Froese & Pauly, 2010), of which only about 1200 are shark and ray species (Heinicke et al., 2009), the potential for interindividual discrimination of bony fishes appears to be considerably underutilised.

BOX 1 **Sources of variability in bony fishes' body markings**

The diversity of body colourations and patterns in bony fishes arises from the spatial arrangement and types of chromatophores in their skin. Different chromatophores (for full list see Sköld et al., 2016) contain distinct pigments or structural elements that, through vertical layering and horizontal arrangement, create various colours and patterns. For instance, xanthophores produce red or yellow pigments (carotenoid and pteridine); melanophores contain melanin for black and brown colours; iridophores have reflective platelets that refract light to create blue hues, silvery tones and iridescent effects (Price et al., 2008; Sköld et al., 2016). Not all fishes possess the same types of chromatophores: zebrafish (*Danio rerio*), for example, have three types (black melanophores, yellow xanthophores and iridescent iridophores, Hirata et al., 2003), whereas others like Nile tilapia (*Oreochromis niloticus*) have four (melanophores, xanthophores, iridophores and erythrophores, Wang et al., 2021). Although blue colouration in most fishes comes from structural iridophores (Bagnara et al., 2007), in mandarin and psychedelic fish (*Synchiropus splendidus*, Figure 1, and *Synchiropus picturatus*), it is pigment based, produced by novel cyanophores (Goda & Fujii, 1995). Together, the arrangement of chromatophores vertically (e.g., stacking yellow xanthophores over blue‐reflecting iridophores to produce green hues, Grether et al., 2004) and horizontally (e.g., contrasting black melanophores next to yellow xanthophores) creates blended colours, gradients and contrasting patterns like stripes or spots (Grether et al., 2004; Sköld et al., 2016). The diversity of fish colourations and patterns thus depends on the number, type and arrangement of chromatophores, allowing for a wide range of combinations through layering and spatial organisation.

The formation of these patterns within fishes, which can range from simple stripes to intricate designs, is governed by one of two main genetic processes (for in‐depth reviews see Kratochwil & Mallarino, 2023; Salis et al., 2019). In the first, genes establish a basic ‘blueprint’ that dictates chromatophore type and distribution across the body. This process, for example, potentially produces the uniform stripes of clownfishes (*Amphiprion* spp.) (Salis et al., 2018, 2021) and cichlids (Hendrick et al., 2019). In the second, genes control the ‘rules’ by which chromatophores interact and disperse across the skin, producing complex patterns such as the stripes of emperor angelfish (*Pomacanthus imperator*) or the contour and spot patterns of ornate boxfish (*Aracana ornata*) (Alessio & Gupta, 2023; Kondo & Miura, 2010; Painter et al., 1999). Irrespective of the underlying mechanism, the potential for individual variation in these patterns will then depend on two key factors: (1) the complexity of the pattern and (2) how tightly its formation is genetically regulated.

First, simple patterns, like the consistent white stripes of clownfishes, vary little across species (Salis et al., 2018), leaving little room for obvious variations within species. Likewise, reduced spot numbers can hinder photo‐identification for Atlantic salmon (*Salmo salar*, Stien et al., 2017). In contrast, species with more complex patterns, such as broad‐barred goby (*Gobiodon histrio*), which feature multiple spots and bands, can show variations in spot size, shape and arrangement, resulting in unique individual markings (Table 1, Wall & Herler, 2009). However, even complex patterns may not always vary; for instance, the thin numerous stripes of emperor angelfish remain consistent in thickness and spacing across individuals (Painter et al., 1999; Salis et al., 2019).

Second, the degree of variation in patterns among individuals will depend on how rigid or flexible the genetic encoding is. This will, to some extent, depend on whether individual variation in markings serves a functional benefit in species (e.g., reducing aggression between conspecifics, facilitating mate choice, see ‘Indirect evidence’, Table 1). More flexible genetic blueprints or rules will allow for deviations due to environmental factors or random developmental fluctuations. This looser genetic control might, for instance, thus result in interindividual differences in stripe number, thickness or regularity or spot placement, size or shape (Kratochwil & Mallarino, 2023). On this logic, tighter control may yield uniform circular spots, whereas more flexibility could result in irregular blotches, which facilitate individual recognition (see Table 1; Figure 1). Additionally, patterns with more elements, like multiple spots, increase the likelihood of obtaining random variation in their occurrence and shape, facilitating individual recognition (assuming the pattern is not tightly regulated) (see Table 1; Figure 1). Thus, the interaction between genetic rigidity and pattern complexity should determine the possibility of individual variation even among genetically similar individuals, much like how identical human twins can have unique fingerprints despite sharing the same DNA (Jain et al., 2002).

In this study, we highlight the potential of photo‐identification for studying and conserving bony fishes. We begin by outlining the advantages of using photo‐identification in fish research and conservation, emphasising its broad applicability and benefits. We then review how photo‐identification has been previously applied to bony fishes, identifying barriers that may explain why it has not yet become a widely used method among fish biologists, conservationists or other potential users. Alongside these barriers, we address potential misconceptions and offer strategies to overcome them, driven by recent technological advancements in camera equipment and artificial intelligence (AI). We also discuss the contexts in which these techniques are most effective and offer recommendations for researchers attempting to apply photo‐identification in their own studies (Box 2). With this review, we therefore aim to encourage the use of photo‐identification as a powerful, non‐invasive tool in bony fish biology and conservation.

BOX 2 **Questions to consider when designing a study that uses photo‐identification to discriminate individual bony fishes**

In general, the appropriateness and likelihood of adopting photo‐identification for bony fishes will primarily depend on what research questions are being asked, as well as characteristics of the focal study species and field/laboratory conditions. Therefore, researchers need to scrutinise their methodological requirements and assess how complimentary these are with the ecology of the species being studied. Broadly, the criteria to consider are as follows:

***Sampling***: How often are individuals being sampled, and how many individuals are being sampled in total? For population‐level metrics, the emphasis lies with capturing as many individuals as possible, whereas behavioural studies may only require a small, consistent cohort of individuals. Are sampling windows affected by external factors (e.g., season, location, time of day)? What sample resolution is necessary, and how does this influence the technology/methods used? Furthermore, is the study organism predictable in time and/or space? For instance, individuals that are small or that exhibit intricate pattern differences may only be suitable for photo‐identification if they have known territories or routines and, therefore, can be captured by high‐resolution camera traps. In addition, how might the methodology account for lateral pattern asymmetry, if present?

***Validation*:** What validation metrics can be used for the study organism? For example, are scars, scratches or limb losses common, or is the species vulnerable to visually conspicuous, stable, external parasites? Would physical tagging be useful on a small cohort to assess the degree of intra‐individual pattern variation? Are there multiple regions that can be used for identification?

***Longevity*:** What is the duration of the study, and how does this compare to the life span of the study organism? How might the mortality of the latter affect the data? How long do you estimate photographic data collection to take, and how long does this compare to the overall study time scale? For instance, mortality will be of interest to those who use photo‐identification for population‐level metrics, whereas frequent individual deaths (and the need to re‐identify new individuals) may significantly influence a short behavioural study. How stable are the body patterns used – will they stay consistent for the period of the study?

***Use of AI*:** Are there enough training data across individuals? Which pattern detection and comparison frameworks are the most suitable for the target species? Can online images of independent individuals help supplement training data? How will AI models be checked for accuracy? How many individuals can be reliably distinguished given the AI/image capture technology being used and the natural variability and diversity in markings of the study species?

## 2 ADVANTAGES OF PHOTO‐IDENTIFICATION FOR RESEARCH AND CONSERVATION OF BONY FISHES

Photo‐identification of individual bony fishes using their unique body markings offers numerous scientific, practical and ethical advantages. Moreover, unlike the elasmobranchs for which individual identification has been applied only in field studies, the ability to distinguish individuals using natural markings is highly relevant for both laboratory‐based studies and for animals maintained in aquaculture facilities.

### 2.1 Scientific benefits

Marshall and Pierce (2012) demonstrate the broad applicability of individual identification for elasmobranch studies, and many of these scientific questions are equally relevant to bony fishes in both field‐ and laboratory studies. From an applied ecological perspective, these include monitoring the size and structure of populations, tracking individuals' movements and residency (e.g., in assessing the use of protected area), monitoring growth rates and identifying life‐history patterns. Individual identification also offers opportunities to quantify individual behaviour within larger groups over time, construct social networks and dominance hierarchies, determine mate choice outcomes, track reproductive efforts and quantify competition between individuals for territories or mates. This opens a range of questions in sociobiology that could not be addressed without information on the identity of individuals. Identifying individuals also allows us to map individual differences in behaviour, or form proxies for fitness. For example, individuals may adopt different strategies for foraging or parental care, and these can be mapped to the survivorship or reproductive success of individuals (both within the field and laboratory). Finally, although we can use individual‐level markings for our own insights into fish sociobiology, such markings could also help us identify and examine the mechanisms fish use to recognise and differentiate between themselves during their social interactions (e.g., Kohda et al., 2024). Overall, individual identification broadens both the scope of the questions in ecology, conservation and behaviour and the resolution at which those questions can be answered.

### 2.2 Research methods benefits

In addition to the range of scientific questions that can be addressed through using individual‐level markings, such approaches can improve research accuracy by allowing us to capture more naturalistic behaviours (as fish will be less affected by stress and disruption) and by reducing sampling biases. For example, capturing individuals to tag them can introduce systematic biases in the sampled population (e.g., bolder individuals may be easier to catch, Wilson et al., 2011). By avoiding the need for capture, photo‐identification could reduce these sampling biases and reduce stress imposed on fishes (see the section Ethical benefits). Additionally, some tagging methods have limited durability, failing after short periods (Sandford et al., 2020) or are unsuitable for specific size classes (Vollset et al., 2020). Tag retention rates can also differ between sexes, further skewing results (Šmejkal et al., 2019). By relying on natural markings, researchers can mitigate against these biases.

### 2.3 Practical benefits

Individual identification can significantly enhance conservation efforts by encouraging public engagement. Citizen science initiatives, for example, can use photo‐identification to mobilise the public to assist in fish monitoring programmes (e.g., ‘Match my Mola’, Nyegaard et al., 2023). These projects raise awareness while providing valuable data for researchers, particularly for monitoring rarer or protected species. In one case, for example, the angling community helped use photo‐identification to provide non‐invasive temporal data for fish monitoring (Kristensen et al., 2020), demonstrating the potential of these methods for large‐scale conservation efforts.

Being able to identify individuals can also encourage the public to engage with conservation efforts by helping them to recognise and follow named animals with distinct personalities and behaviours (Manfredo et al., 2020). For example, the famous Napoleon wrasses (*Cheilinus undulatus*), ‘George’ and ‘Wally’ in Australia and ‘Frank’ in the Cook Islands have helped foster public interest in the species. Likewise, the ‘Coral City’ underwater streaming project also exemplifies how technology can deepen public engagement by allowing viewers to follow specific animals in real time, thereby benefitting species protection by increasing public empathy and concern for their welfare (Hearne, 2007).

Another important practical application of individual recognition is in law enforcement, particularly for species protected under international regulations. Unique body patterns can serve as a non‐invasive method to track specific animals and identify those involved in illegal trade. For example, using body pattern recognition, authorities could better monitor endangered species like Napoleon wrasse (*C. undulatus*), which are often illegally imported into and retailed in seafood markets like Hong Kong (Hau & Sadovy de Mitcheson, 2019). These imported fish are not individually tagged, and therefore, photo‐identification methods could enhance efforts to track and regulate the trade of endangered species more effectively (e.g., Hau et al., 2025).

Finally, there is a growing shift within aquaculture from mass production and population‐level management towards a focus on individual‐level traits in line with the ‘Precision Livestock Farming’ framework. This individual‐level approach enables managers to identify, monitor and select for desirable traits, tailor feeding regimes and track growth, parasite load and disease resistance/progression (Macaulay et al., 2021). Ultimately, these individual‐level insights can improve fish welfare (Schraml et al., 2020) and support the efficiency of more selective breeding efforts (Farias et al., 2017), thereby raising both the economic and ethical standards of the industry. Although traditional tagging methods are often applied to a subset of the population, such as ‘sentinel’ individuals (e.g., Macaulay et al., 2021), non‐invasive identification of all individuals (e.g., via iris or body pattern recognition: Merz et al., 2012; Castillo, 2018; Schraml et al., 2020) has the potential to extend precision farming approaches across intensive aquaculture operations.

### 2.4 Ethical benefits

Using natural body markings to identify individuals presents ethical advantages over traditional tagging methods. Although newer tagging techniques, such as visible implant elastomers (VIE) or photonic marking, are effective, they still require invasive procedures, including anaesthetising animals for tag implantation. Furthermore, VIE tags are not permanent and may be lost over time. Other non‐invasive approaches, such as skin markers (Branconi et al., 2019), last only a few days and still require handling the fish. External electronic tags, meanwhile, can affect survival, cause tissue damage, reduce mobility or increase predation risk in some species (Jepsen et al., 2015). In countries such as the UK, United States and Australia, invasively marking bony fishes therefore requires licences. Using natural markings, however, aligns with the ‘Refinement’ principle of the 3Rs (replacement, reduction and refinement) by minimising stress and harm to the animals (Sneddon et al., 2017). Thus, identifying individuals through their natural markings can reduce the necessity to physically tag individuals, accelerating research and increasing the likelihood of capturing naturalistic behaviours and responses under both field and laboratory conditions. This method is therefore especially useful for fishes that are hard to capture, protected from capture or whose behaviour might change due to handling or tagging.

In laboratory and aquaculture settings specifically, the ability to consistently distinguish individuals over time offers important logistical and husbandry benefits. These include reducing the need for social isolation in individual‐level studies, enabling accurate assignment of food, treatments and enrichment per individual, and improving the efficiency and welfare of long‐term monitoring and care. Indeed, individual identification is variously applied in aquaculture to improve health treatment and facilitate handling and tracking of individuals over time; aquaculture facilities have found that natural markings allow for more efficient and safer fish handling (Zion, 2012).

## 3 CURRENT APPLICATION OF PHOTO‐IDENTIFICATION IN BONY FISHES

The concept of distinguishing individual bony fish by their unique body markings dates back to the 1990s, occurring in parallel to its early use with elasmobranchs (e.g., Lelong, 1999; van Rooij et al., 1996), with evidence of even earlier applications (e.g., Dubin, 1981). However, it is only in the last 15 years, or so, that photo‐identification based on body markings has gained greater attention and application in bony fish research (e.g., Table 1). Despite initial scepticism regarding the permanence and distinctiveness of these markings (Calò et al., 2013; Dala‐Corte et al., 2016), advancements in camera technology, computer vision and AI, particularly digital imaging in the mid‐1990s and facial recognition software in the early 2000s, have made it easier to identify individuals accurately and track them over time. Automation of photo‐identification has been partly driven by advancements in aquaculture where, for example, it is beneficial to create individualised treatments, track biomass and monitor individual fish's health and condition over time (Cisar et al., 2021; Zion, 2012).

**FIGURE 2:**
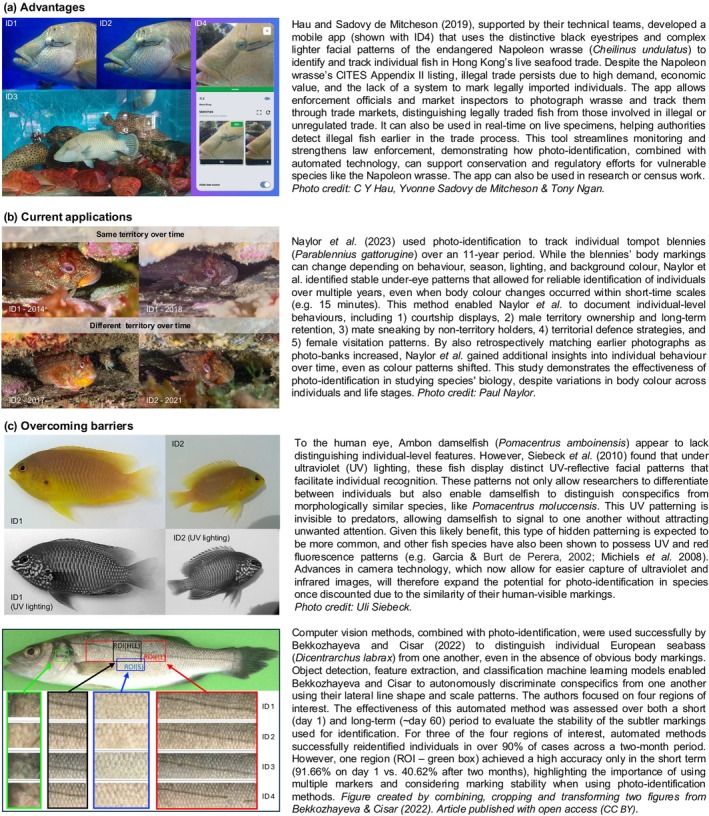
Three case studies highlighting the conservation advantages of photo‐identification (a), its applications to understanding bony fish ecology (b) and its use after overcoming previous technological barriers (c).

Although the adoption of photo‐identification methods for bony fishes has been slower than for elasmobranchs and other megafauna, growing conservation concerns, particularly for threatened and iconic species, have helped encourage interest in developing non‐invasive identification techniques. Ethical considerations and limitations relating to the invasive tagging of individuals have further driven the need for such methods. As a result, studies have used spots, blotches, contours or stripes to investigate key aspects of bony fish biology, including population sizes, habitat use and behaviours (Table 1; Figures 1 and 2b). Unique body markings have also been employed to combat illegal wildlife trade, such as in the case of a large wrasse (Figure 2a, Hau & Sadovy de Mitcheson, 2019; Hau et al., 2025). Moreover, even fishes lacking obvious body markings can now be individually identified through features such as scale patterns and lateral‐line shapes (Figure 2c, Bekkozhayeva & Cisar, 2022; Huntingford et al., 2013). Given the diverse colour patterns of bony fishes in reef environments (Salis et al., 2019), one might expect photo‐identification studies to focus predominantly on reef‐associated species. Surprisingly, this is not the case for fishes, with nearly as many temperate species, for example, listed in Table 1 (which is not an exhaustive list). Additionally, despite the wide variety of patterning in freshwater species, most studies appear to focus on marine fishes.

In a parallel, yet indirectly related, field researchers are exploring whether individual recognition based on body markings plays a functional role in fishes' social interactions, which could expand photo‐identification's applicability across species. Fishes that engage in frequent repeated interactions, such as those involved in mate selection, territorial disputes or cooperative breeding, may benefit from recognising specific individuals. Indeed, some species (e.g., Table 1) use certain pattern features to differentiate familiar from unfamiliar individuals, potentially reducing the costs associated with aggression (e.g., Kohda et al., 2015; Saeki et al., 2018). This functional importance of individual‐level markings could ultimately increase the selection pressure for varied individual body markings for certain species (Box 1), further enhancing the potential for photo‐identification across bony fishes. Species with strong social structures, such as territorial or cooperative fishes where individual recognition could be beneficial for conspecific interactions, may thus be particularly well suited for photo‐identification studies (e.g., Figure 2b, Naylor et al., 2023; Naylor & Jacoby, 2016). However, even in fishes that apparently lack complex social behaviours (e.g., shoaling species), where the benefits for fish of discriminating among conspecifics are less obvious, individual recognition abilities via the use of fine‐scale facial differences have still been demonstrated (e.g., guppy, *Poecilia reticulata*: Sogawa et al., 2023, and medaka, *Oryzias latipes*: Wang & Takeuchi, 2017). The subtle features used by these fishes may become more apparent to researchers through recent technological advances [e.g., ultraviolet (UV) imaging, machine learning; see ‘Barriers’ below], expanding the applicability of photo‐identification to a much broader range of species, both in the laboratory and the field.

## 4 BARRIERS AND STRATEGIES FOR IMPLEMENTING PHOTO‐IDENTIFICATION FOR BONY FISHES

There are two primary assumptions to meet when using photo‐identification to study individual bony fishes, as outlined by Marshall and Pierce (2012): first, individuals must be distinguishable from one another in a non‐subjective manner, and second, they must be re‐identifiable over relevant time frames. This means that suitable species for photo‐identification should possess individually distinct, stable markings. Although these assumptions are readily met in many elasmobranch (Marshall & Pierce, 2012) and cetacean (Urian et al., 2015) species, applying photo‐identification to bony fishes presents some similar yet additional challenges. Together, these challenges may have limited its wider use in bony fish research. Indeed, the use of photo‐identification for bony fishes has been previously argued to be ‘impractical’ due to the ‘difficulty in finding permanent and identifiable marks’ (Calò et al., 2013). In this study, we discuss the challenges in using photo‐identification in bony fishes and offer solutions that could help overcome them to promote the use of photo‐identification for fishes.

### 4.1 Identifiable marks

#### 4.1.1 Challenge

In contrast to elasmobranchs, which display a wide array of striking markings, such as individual fingerprint‐like patterns and complex spot patterns (Marshall & Pierce, 2012), many bony fishes may initially seem to lack obvious features for distinguishing among individuals. This perceived limitation could hinder researchers' confidence in attempting to make individual comparisons.

#### 4.1.2 Strategies to overcome challenge

Although many reef fishes display a wide variety of colours and patterns, ranging from simple spots and stripes to more intricate labyrinthine arrangements (Salis et al., 2019), making them strong candidates for marking‐based individual identification, the assumption that many other fishes do not show identifiable marks is likely misconstrued. Although 30% of 18,114 freshwater and marine fishes (across 559 families) have been characterised by having monotone patterns, the rest exhibit one of nine other distinct pattern types (Miyazawa, 2020), each of which could facilitate interindividual differences to varying degrees, depending on the pattern's complexity and the level of genetic control (see Box 1). Indeed, 23% of these fishes sampled show blotched patterns (Miyazawa, 2020), a feature previously used to distinguish individual fish (e.g., Lelong, 1999; Martin‐Smith, 2011, Table 1; Figure 1). Obvious colouration patterns may also not be necessary for photo‐identification: medaka (*O. latipes*), for example, appear to be able to individually discriminate between conspecifics without any obvious visual patterns to human observers (Wang & Takeuchi, 2017). Fewer subtle differences in patterning/appearance, and/or those that are invisible to the human eye, could now be detected and captured using advances in camera technology. For example, Ambon damselfish (*Pomacentrus amboinensis*) possess individually unique UV reflective facial patterns, which are not visible to humans without UV light (Figure 2c, Siebeck et al., 2010). Thus, given the prevalence of diverse colouration patterns in fishes, and the ability of some fish species to discriminate between conspecifics without obvious patterns, more examples of interindividual variation are expected (see Box 1 for limitations and considerations).

Although we focus heavily on body colourations (e.g., Box 1), other physical features such as body scars (e.g., *Epinephelus itajara*, Giglio et al., 2014), the arrangement of bony plates (e.g., *Rineloricaria aequalicuspis*, Dala‐Corte et al., 2016) and scales (Figure 2c, Bekkozhayeva & Cisar, 2022; Huntingford et al., 2013) or the presence of ectoparasites (e.g., black spot syndrome, Kohl et al., 2019) can also serve as distinguishing characteristics for certain species (but see ‘Stability’ and ‘Validation’ below). Advances in AI have now made it significantly easier to recognise subtle differences between and among individuals. Indeed, such advances may allow us to discriminate between individuals with seemingly plain bodies. For example, computer vision techniques have allowed researchers to successfully discriminate among conspecifics of European seabass (*Dicentrarchus labrax*, Figure 2c) and common carp (*Cyprinus carpio*) based on scale patterns and lateral‐line shapes (Bekkozhayeva & Cisar, 2022). As computing power and AI technologies continue to improve, the potential for utilising a broader range of physical traits for individual identification (Pérez‐Escudero et al., 2014; Romero‐Ferrero et al., 2019), depending on the species and objectives (Box 2), will only increase.

### 4.2 Stability of markings

#### 4.2.1 Challenge

As with other taxa, the colouration of many bony fishes can change over time (Leclercq et al., 2010; Sköld et al., 2016), which presents challenges for reliable photo‐identification. One issue is that many species can rapidly alter their colouration through physiological changes (Sköld et al., 2016). These shifts in body lightness or darkness can impact the visibility and contrast of markings (Mills & Patterson, 2009; Sugimoto, 2002) and may even distort certain features, such as the orientation of stripes (e.g., *Upeneichthys lineatus*, Tosetto et al., 2024). In more extreme cases, fish can completely transform their appearance, altering both the shape and placement of their patterns. For example, the slender filefish (*Monacanthus tuckeri*) can switch among 16 different pattern features within 1–3 s, significantly altering its appearance to blend with its surroundings (Allen et al., 2015). Similarly, fishes that mimic other species can rapidly change between different colour morphs (Cortesi et al., 2015; Côté & Cheney, 2005). Such dynamic changes present significant obstacles to reliable photo‐identification.

In addition to rapid shifts in colouration, many fishes undergo gradual morphological changes in their colouration over time, often due to shifts in chromatophore number, morphology or pigment content (Leclercq et al., 2010; Sköld et al., 2016; Sugimoto, 2002). For example, juveniles may have markings, such as stripes, which fade or disappear as they mature (Salis et al., 2018), whereas other species, such as those in the *Pomacanthus* genus, develop additional stripes as they grow (Kondo & Miura, 2010). Seasonal changes in ornamental colouration are also common; male cichlids, temperate blennies (Figure 2b) and many reef fishes adopt different colour patterns during breeding seasons (Kodric‐Brown, 1998; Miyagi & Terai, 2013). More extreme shifts are seen in coral reef species as individuals transition from juvenile to adult stages, or change their sex as sequential hermaphrodites, often changing both their colour palettes and patterns (Cardwell & Liley, 1991; Hodge et al., 2020).

#### 4.2.2 Strategies to overcome challenges

The importance of marking stability in focal study species will ultimately depend on the scale and objectives of the study (Box 2). In long‐term research, examining life‐history strategies or population dynamics, it is crucial that individuals can be re‐identified over extended time periods, necessitating that their markings remain stable across years or even lifetimes. For longer‐term studies where there are high turnovers of individuals, it could still be feasible to use photo‐identification, provided that individual fish can be reliably distinguished through repeated sampling during the study period (e.g., Figure 2b). In contrast, shorter‐term studies may be less reliant on long‐term stability if individuals can be reliably distinguished within the study's timeframe. Ultimately, the time scale over which markings remain stable should align with the research questions and nature of the study.

With sufficient knowledge of a species' life history, the timing of when markings will change (particularly over ontogenetic shifts) can often be predicted. This predictability therefore helps with photo‐identification when the study focuses on specific life stages (e.g., van Rooij et al., 1996). Indeed, even with species that show highly dynamic colour changes, such as for sunfish (*Mola alexandrini*, Table 1), which rapidly change the contrast of their patterns from low contrast to bold display, individual identification can still be possible through video capture (see Nyegaard et al., 2023). Moreover, long‐term monitoring allows researchers to retrospectively analyse earlier photographs of individuals. By comparing them with updated information on different pattern occurrences, individuals can be re‐identified over time despite, for example, behaviourally mediated pattern changes (e.g., as in Naylor et al., 2023, Figure 2b). Nevertheless, some species with highly dynamic and unpredictable colouration changes may still be unsuitable for photo‐identification. With the continual accrual of detailed species‐specific ecological and morphometric knowledge, details such as the longevity or the site fidelity of a species, and the permanence and stability of different patterns or features, will guide the choice of suitable study species, pattern features, study locations and research questions (Box 2).

### 4.3 Capturing data

#### 4.3.1 Challenge

Successful photo‐identification can often require capturing high‐resolution images of an individual's body markings, with the resolution of these markings perhaps varying with an individual's body size. This process is thus generally easier for larger, long‐lived, slow‐growing animals like manta rays (Marshall et al., 2011), humpback whales (Barlow et al., 2011) and whale sharks (Arzoumanian et al., 2005), which are also often solitary or found in small groups. These species often interact with the water surface and move relatively slowly, enabling researchers to collect data without entering the water. Although using natural markings can be a cost‐effective approach, scaling it up for larger studies may also increase overall costs.

In contrast, bony fishes present unique challenges due to their larger, denser populations that often overlap within a single underwater habitat. Getting close enough to capture high‐resolution images can also provoke flight or aggressive responses in some species (Andradi‐Brown et al., 2018; Stamoulis et al., 2019). Although reef‐dwelling fishes in shallower waters are more accessible and facilitate repeat encounters with the same individuals, species found in pelagic, deep‐sea or turbid freshwater environments are more difficult to observe. Moreover, many suitable bony species for photo‐identification are social (Negro et al., 2020), making real‐time identification and tracking of specific individuals challenging in many species. Additionally, many fishes have lateral differences in body patterning (e.g., Hau & Sadovy de Mitcheson, 2019; Levy et al., 2014), meaning that photographs of both sides of the fish should ideally be captured for thorough comparison. Therefore, capturing usable photographs for photo‐identification in bony fishes is often more difficult compared to larger elasmobranchs and cetaceans.

#### 4.3.2 Strategies to overcome challenges

Advances in camera technology have improved image resolution, making it possible to capture finer, complex or subtle markings on smaller and fast‐moving fish. Additionally, the development of unmanned underwater cameras, such as remote cameras and camera traps (Dunkley et al., 2023; Favaro et al., 2012; Purser et al., 2020; Williams et al., 2014), has allowed researchers to capture footage without triggering avoidance behaviours in target species. Continuing improvements in battery technology also enable extended camera deployments on sampling schedules (e.g., Dunkley et al., 2023; Mouy et al., 2020). More confined conditions, such as small ponds, aquaculture pens, laboratory or aquaria, also lend themselves well to the use of photo‐identification techniques where the circumstances for photo‐taking can be optimised.

Where cost was once prohibitive, high‐quality cameras are also becoming more accessible and affordable. Compact digital underwater cameras, action cameras (e.g., GoPro) and mobile phone–based cameras are now widely owned by the public, allowing for the collection of footage over larger spatial and temporal scales at a lower cost and with greater ease while promoting citizen science initiatives (Roberts et al., 2022; Struthers et al., 2015). Novel low‐cost, low‐power camera options, such as those developed by Raspberry Pi, are also gaining popularity for remote camera deployments (e.g., Dunkley et al., 2023; Mouy et al., 2020). SCUBA diving technology has also advanced, with new equipment such as rebreathers becoming more accessible to recreational and scientific divers. Rebreathers minimise the noise generated by diving regulators, allowing for closer filming distances during surveys (Andradi‐Brown et al., 2018), which is especially useful for capturing detailed images of individuals with minimal disturbance.

Additionally, computer‐assisted‐ and AI techniques can significantly enhance the speed and accuracy of photo‐identification's image processing, especially as image and individual fish sample sizes increase. AI systems can automate pattern recognition and individual identification (e.g., Figure 2c), as demonstrated in studies on elasmobranchs and cetaceans (Hughes & Burghardt, 2017; Renò et al., 2020). For example, the commonly used Interactive Individual Identification System (I3S, https://reijns.com/i3s/) efficiently assists in pattern recognition across various species requiring limited human input and zero coding due to the built‐in algorithms (Calmanovici et al., 2018; Correia et al., 2021; Hook et al., 2019; McInnes et al., 2020; Rocha et al., 2013; Russo & Loy, 2020; Sannolo et al., 2016). Continued developments and applications of AI methods (e.g., Patton et al., 2023) will facilitate the capturing of data for the use of photo‐identification for bony fishes further.

### 4.4 Validating markings

#### 4.4.1 Challenge

Reliable photo‐identification depends on demonstrating that the body features used to distinguish among individuals remain consistently different across them. In larger species like elasmobranchs and cetaceans, scars, such as rake marks or missing fin chunks (e.g., Penketh et al., 2021), often serve as reliable validation tools for identifying individuals. However, such prominent, long‐lasting markers may be less common in bony fishes (though some exceptions exist, e.g., Bekkozhayeva & Cisar, 2022; Giglio et al., 2014; Reist et al., 1987; Rosen & Hales, 1980). Injuries that frequently cause this scarring in larger marine animals, such as boat propeller strikes, net entanglements (Herr et al., 2020; Penketh et al., 2021) or severe conspecific aggression (MacLeod, 1998; Ritter & Amin, 2019), are more likely to be fatal for many fishes (especially smaller individuals). Additionally, bony fishes can often regenerate lost or damaged tissues, such as fins and scales (Nakatani et al., 2007), making scars and other damage less permanent and reliable for identification.

#### 4.4.2 Strategies to overcome challenges

Although scars may be used for fishes (where applicable), certain morphological and ecological traits can also provide alternative validation techniques. Indeed, head shape was used to discriminate between seahorse individuals (Table 1, Correia et al., 2021). Likewise, long‐term external parasites, such as blackspot trematode infections, for example, can leave distinct dermal spots on some fishes (Hoffman & Putz, 1965; Kohl et al., 2019; Teixeira‐de Mello & Eguren, 2008), potentially serving as useful validation markers for vulnerable species. For site‐attached species, such as sedentary cleaner fishes (e.g., *Elacatinus* spp.), which inhabit specific coral structures (Dunkley et al., 2019; Whiteman et al., 2002; Whittey et al., 2021), or highly territorial species (e.g., *Parablennius gattorugine*, Naylor & Jacoby, 2016), spatial discrimination could aid in validating individual markings. However, in such cases, where location alone may be enough to distinguish individuals in the short term (e.g., Dunkley et al., 2019), the need for individual discrimination through body markings in the first place depends on the study's context and timeframe (see Naylor et al., 2023; Naylor & Jacoby, 2016 for an example where this is useful, Figure 2b). On a larger scale, comparing body marking patterns between geographically distinct, independent populations of reef fishes could also help determine which pattern features are fixed and which are flexible. For example, in white‐spotted filefish (*Cantherhines macrocerus*), the location of certain spots appears consistent among individual photographs from independent, geographically separated populations (as observed via ‘Google Images’), where movement between regions is highly unlikely (Dunkley, personal observation). This can also be highlighted in Figure 1 where different markings are observed between independently obtained photos within a species. Additionally, individual‐level markings can be validated by tagging a small cohort (Box 2) or through genetic techniques (Monteiro et al., 2014).

As a bottom line, to validate markings, it is beneficial to identify an additional body feature that also serves as an individual‐level marker. The combined effect of multiple markings increases confidence that one individual can be successfully distinguished from another. Although these supplementary features may not serve as permanent markers, they can still provide valuable validation for reliably distinguishing individuals. The success of these methods will therefore largely depend on study duration and the stability of the chosen markers over time (e.g., Figure 2c).

## 5 CONCLUSION

As we outline here, despite the remarkable diversity of bony fishes and the potential for using their natural body markings for individual‐level identification, photo‐identification methods appear remarkably underutilised compared to their application for other aquatic species, such as cetaceans, sharks and rays. Yet, this non‐invasive approach offers considerable promise in field, laboratory and commercial environments, especially in situations where traditional methods for discriminating between individuals (e.g., marking or tagging techniques, or social isolation) are impractical, ethically and logistically problematic and/or risk altering behaviour. Through individual recognition, photo‐identification can help fill critical knowledge gaps across fish biology (e.g., tracking individual growth rates and quantifying population sizes), ecology (e.g., quantifying movement patterns and social interactions) and conservation (e.g., monitoring and enforcing regulations around fish trade) while also addressing key ethical and logistical challenges. Although misconceptions and practical challenges may have previously limited the use of this method for bony fishes, here we have provided practical solutions and considerations to overcome these barriers. Recent advancements in camera technology and computer vision, for example, now make it increasingly feasible to automate individual recognition and also detect subtle, previously unobservable variations in appearance. These computational developments also expand the spatial and temporal resolution with which we can monitor individual fish across diverse habitats and study designs. Ultimately, increasing the uptake of photo‐identification in bony fish research can enhance our capacity to monitor populations, inform management decisions and support the conservation of ecologically and economically important species across both marine and freshwater systems.

## AUTHOR CONTRIBUTIONS

All authors contributed to the conceptulisation and preparation of the manuscript. Katie Dunkley led the editing and revision of the manuscript.

## FUNDING INFORMATION

Katie Dunkley was supported by the Christ's College's Charles Darwin and Galapagos Islands Fund. Samuel R. Matchette and Roxanne B. Holmes were supported by the Whitten Programme in Tropical and Aquatic Biology. James E. Herbert‐Read was supported by the Whitten Lectureship in Marine Biology. Christian Drerup was supported by a Natural Environment Research Council (NERC) C‐CLEAR PhD studentship (NE/S007164/1) and a Cambridge Trust European Scholarship. The University of Hong Kong, the Ocean Park Conservation Foundation Hong Kong (FH02.2021) and an anonymous donor variously supported work on Napoleon wrasse (Yvonne Sadovy de Mitcheson and Cheuk Yu Hau).
